# Prediction of Cell Migration in MDA-MB 231 and MCF-7 Human Breast Cancer Cells Treated with *Albizia Lebbeck* Methanolic Extract Using Multilinear Regression and Artificial Intelligence-Based Models

**DOI:** 10.3390/ph16060858

**Published:** 2023-06-08

**Authors:** Huzaifa Umar, Nahit Rizaner, Abdullahi Garba Usman, Maryam Rabiu Aliyu, Humphrey Adun, Umar Muhammad Ghali, Dilber Uzun Ozsahin, Sani Isah Abba

**Affiliations:** 1 Operational Research Centre in Healthcare, Near East University, TRNC Mersin 10, Nicosia 99138, Turkey; 2 Biotechnology Research Centre, Cyprus International University, TRNC Mersin 10, Nicosia 99258, Turkey; 3 Department of Analytical Chemistry, Faculty of Pharmacy, Near East University, TRNC, Mersin 10, Nicosia 99138, Turkey; 4 Department of Energy System Engineering, Cyprus International University, TRNC, Mersin 10, Nicosia 99258, Turkeyhadun@ciu.edu.tr (H.A.); 5 Department of Medical Biochemistry, Faculty of Medicine, Near East University, TRNC, Mersin 10, Nicosia 99138, Turkey; 6 Research Institute for Medical and Health Sciences, University of Sharjah, Sharjah P.O. Box 27272, United Arab Emirates; 7 Department of Medical Diagnostic Imaging, College of Health Sciences, University of Sharjah, Sharjah P.O. Box 27272, United Arab Emirates; 8 Interdisciplinary Research Centre for Membranes and Water Security, King Fahd University of Petroleum & Minerals, Dhahran 31261, Saudi Arabia

**Keywords:** metastasis, breast cancer cells, artificial neural network, adaptive neuro-fuzzy inference system, multilinear regression analysis

## Abstract

Breast cancer is a common cancer affecting women worldwide, and it progresses from breast tissue to other parts of the body through a process called metastasis. *Albizia lebbeck* is a valuable plant with medicinal properties due to some active biological macromolecules, and it’s cultivated in subtropical and tropical regions of the world. This study reports the phytochemical compositions, the cytotoxic, anti-proliferative and anti-migratory potential of *A. lebbeck* methanolic (ALM) extract on strongly and weakly metastatic MDA-MB 231 and MCF-7 human breast cancer cells, respectively. Furthermore, we employed and compared an artificial neural network (ANN), an adaptive neuro-fuzzy inference system (ANFIS), and multilinear regression analysis (MLR) to predict cell migration on the treated cancer cells with various concentrations of the extract using our experimental data. Lower concentrations of the ALM extract (10, 5 & 2.5 μg/mL) showed no significant effect. Higher concentrations (25, 50, 100 & 200 μg/mL) revealed a significant effect on the cytotoxicity and proliferation of the cells when compared with the untreated group (*p* < 0.05; *n* ≥ 3). Furthermore, the extract revealed a significant decrease in the motility index of the cells with increased extract concentrations (*p* < 0.05; *n* ≥ 3). The comparative study of the models observed that both the classical linear MLR and AI-based models could predict metastasis in MDA-MB 231 and MCF-7 cells. Overall, various ALM extract concentrations showed promising an-metastatic potential in both cells, with increased concentration and incubation period. The outcomes of MLR and AI-based models on our data revealed the best performance. They will provide future development in assessing the anti-migratory efficacies of medicinal plants in breast cancer metastasis.

## 1. Introduction

Breast cancer (BCa) is one of the utmost malignancies affecting women. It is the second leading cause of death in cancer patients, accounting for approximately 24% of cancer-associated cases [1]. About 50% of women with BCa cases usually develop metastasis in their bones and ultimately die from the development of the disease at these sites. Cancer is the second disease leading to an increase in mortality rate after cardiovascular diseases. It originates from the uncontrolled growth of the cells, proliferating and invading other body tissues through a process called metastasis [2].

Metastasis involves the movement of uncontrollable growth cells from one site to another [3]. Various physiological and pathological processes across several biology disciplines, including cancer, wound healing, inflammation, cell growth and differentiation, depend on it [4,5]. The migration of cells in wound healing occurs in various cells, including fibroblast, keratinocytes, endothelial cells and some unique macrophages type that promotes growth factors [6]. Metastasis is the process by which tumour cells migrate from primary sites to other parts of the body, and it is the fundamental difference that differentiates benign and malignant tumours [7]. The spread of tumour cells to areas far away from the primary tumour occurs through the invasion of lymph, blood, or body spaces to distant organs, to physical obstruction [8,9]. The process of invasion and metastasis involves a variation in cell interactions; some cells do not have the ability to metastasize, and the interaction of tumour cells with the associated stroma affects the ability of cancer cells to metastasize [10]. Complex mechanisms involved in cancer metastasis offer specific targets for the progress of anti-metastatic therapies [11].

Medicinal plants can produce different chemical compounds that carry out specific biological functions and protect the plants against predators; *Albizia lebbeck* is among those plants [12]. *Albizia lebbeck* is otherwise known as a ‘flea’ or ‘siris’ tree; some call it fry wood. The plant belongs to the Fabaceae (Leguminosae) family, Mimosoideae subfamily, mainly grows to a height of 18–30 m with a trunk width of 50 cm, has bipinnate leaves with (1–) 2–4 (−5) pairs of pinnae, turns a deep yellow colour during the dry season, flowers pedicels 1.5–4.5 mm long, has stamens of about 7.5–15 cm long and seed pods 12–35 cm long, 3–6 cm wide that contain 6–12 seed of varies length ranging between 15–30 cm [13,14,15]. Various parts of the tree, including the root, stem and leaves, are used as ethnomedicine, ornaments, coffee and tea, and the bark is used for making soap [16].

The *A. lebbeck* flower from the Saudi Arabia region, extracted using dichloromethane with 71.6% yield, showed anti-inflammatory activity. This study suggests the use of the plant in treating inflammation and recommends isolating the components responsible for the anti-inflammatory activity [17]. Saponins are the major phytochemical constituents found on the flower of the plant, and the two types of saponins named *lebbeckosides* A and B isolated from revealed significant cytotoxic activity on glioblastoma stem-like TG1 human and glioblastoma U-87 MG cells lines. [18]. Biosynthesized zinc oxide nanoparticles using various concentrations of *A. lebbeck* aqueous extract as a chelating agent revealed antioxidant, antimicrobial and cytotoxic activity against two distinct human breast cancer cells [19].

Several studies were conducted on the linear approach and neural network models for biomedical applications. For example, Hasan reported the application of artificial neural networks (ANN) to be an effective technique that can be utilized in the field of diagnosis to reduce the negative menace caused by breast cancer. Chances of survival are comparatively high if detected at an early stage [20]. Pan et al. employ a reliable detection tool for the elucidation of tumour cells as well as for the biodistribution of anti-tumour agents within the entire body to fully understand the mechanism to be used in treating cancer metastasis. Deep learning-based quantification was employed in a spontaneous metastasis model for human breast cancer. The result proved to have shown the reliability of imaging technologies for detecting cancer cells [21]. Liu et al. also revealed a trend of number growing dependence on microarray data and protein biomarkers. More importantly, machine learning is equally improving the fundamental understanding of cancer development and progress [22]. Sadoughi et al. stated that breast cancer is a common cancer that affects women globally. Even though various medical progress has been achieved, this disease is the second major deadly disease in the world. Thus, diagnosis in the early stage of this disease plays a role in minimizing the mortality rate. Even though it is usually challenging to diagnose anomalies in breast cancer, various devices such as thermography, mammography and ultrasound can screen for breast cancer. In this regard, computational models assist in investigating chest changes using artificial intelligence (AI) and image processing tools [23]. Wang et al. combine deep learning system ideas with human pathologists’ ideas to elucidate breast cancer diagnosis. This combination is proven to have shown an 85% reduction in human error rate for the prediction and diagnoses of breast cancer and shows efficient improvements in the accuracy and precision of pathological diagnoses [24]. 

Regarding the studies mentioned above, there are insufficient applications of AI-based models such as ANFIS, which served as the universal hybrid approximate that combined the power and concept of both neural networks and fuzzy logic. Hence, the principal motivation of this research is presented on the basis of comparative analysis between the non-linear models’ ANN and ANFIS with the classical linear models MLR in the prediction of cancer metastasis (cell migration) on human BCa cells (MDA-MB 231 and MCF-7). To the author’s best knowledge, this study is the first to elucidate the comparative analysis of these models in cells treated with medicinal plants.

In this study, the cytotoxicity, anti-proliferation and anti-migratory potential of various concentrations of *A. lebbeck* methanolic (ALM) extract on MDA-MB 231 and MCF-7 cells were studied. Furthermore, we employed and compared an artificial neural network (ANN) and an adaptive neuro-fuzzy inference system (ANFIS), which are artificial intelligence base models, and a multilinear regression analysis (MLR) non-linear model; we predict cell migration as an initial stage to detect metastasis on the treated cancer cells using our experimental data.

## 2. Results and Discussion

### 2.1. Experimental Results

In this present study, the total phenolic content (TPC) and total flavonoid content (TFC) present in ALM extract are 8177.88 ± 20.52 GAE µg/g and 2177.35 ± 19.71 QE µg/g, respectively. Phenolic compounds present in plants are the most important antioxidant that can terminate free radicals and their bioactivity, which is facilitated by a hydroxyl group in their terminal chain [25]. Studies proved TPC to be very effective in scavenging various classes of oxidizing molecules, such as free radicals generated during lipid peroxidation [26]. In addition, studies revealed that flavonoids have medicinal properties with variable structures of phenolic compounds, and they are found in stem bark, fruits, vegetable, roots, flowers and tea [27].

**Figure 1:**
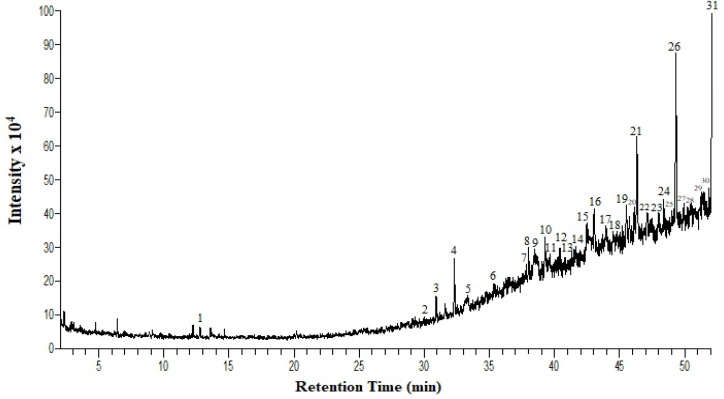
GC-MS chromatogram of *Albizia lebbeck* methanolic extract bioactive compounds. The numbered peaks correspond to the numbers and molecules in Table 1.

GC-MS analysis of the extract revealed some compounds that are of medicinal value; they could be responsible for the anti-metastatic potential. The biological activities of the compounds detected are summarized in Table 1, and the chromatogram peaks illustrated in Figure 1 correspond to the numbers and molecules listed in the table. Major compounds identified with anti-cancer activity from our extract were hexadecanoic acid, quercetin 7,3’,4′-tri methoxy, 2-propenoic acid, tetradecyl ester, 9,12,15-octadecatrienoic acid, 1-hexadecanol, 2-methyl- (CAS) and trans-2-phenyl-1,3-dioxolane-4-methyl octadec-9,12,15-trienoate. The effect of the extract on proliferation and lateral motility in the cells might be due to the presence of the above-identified compounds, which might be responsible for the anti-metastatic potential of the plant, and it could be a promising cause for the prevention of cancer metastasis.

**Figure 2:**
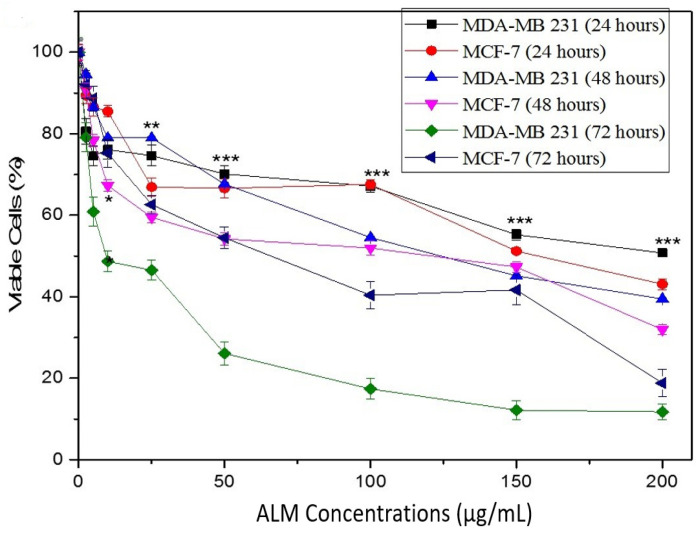
Effect of *Albizia lebbeck* methanolic (ALM) extracts concentrations on the viability of MDA-MB 231 and MCF-7 breast cancer cell lines. The cytotoxic effects of the extracts were studied using a trypan blue exclusion assay and observed after a 24, 48 and 72 h incubation period. Significant differences ((*) *p* < 0.05; (**) *p* < 0.01 and (***) *p* < 0.0001) observed in our graph are applied to all treatments with the same concentration. Data are presented as mean ± SEM of at least replicates experiments (*n ≥* 3) and analyzed using one-way ANOVA followed Newman-Keuls post hoc analysis.

The cytotoxic potential of ALM extracts performed using a trypan blue assay was used as initial findings revealed the concentrations of the extract that do not affect the viability of the cells. The results showed significant differences with higher concentrations of ALM in both treated cells as compared with the untreated cells (*p* > 0.05, *n* ≥ 3, Figure 2). Studies revealed that isolated saponin derivatives from the stem bark of *Albizzia Julbrassin* and *Albizia subdimidiata* butanolic extract had shown cytotoxic effects against human breast cancer (MCF-F), human alveolar basal epithelial (A549) and human colon adenocarcinoma [39]. Furthermore, cytotoxic potential against MDA-MB 231 breast cells was observed following treatment with biosynthesized zinc oxide nanoparticles using *Albizia lebbeck* extract, which resulted from the participation of the phytochemicals present in the plant stem bark [19].

**Figure 3:**
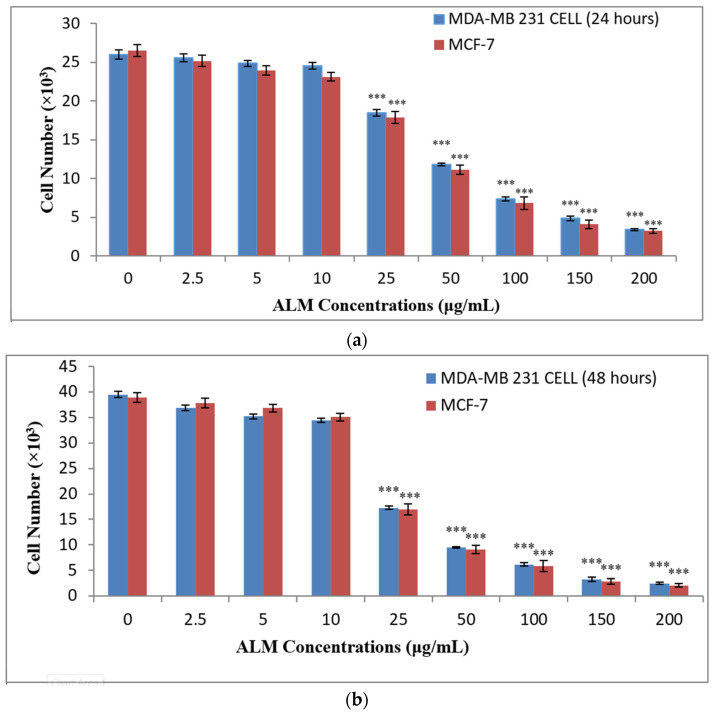

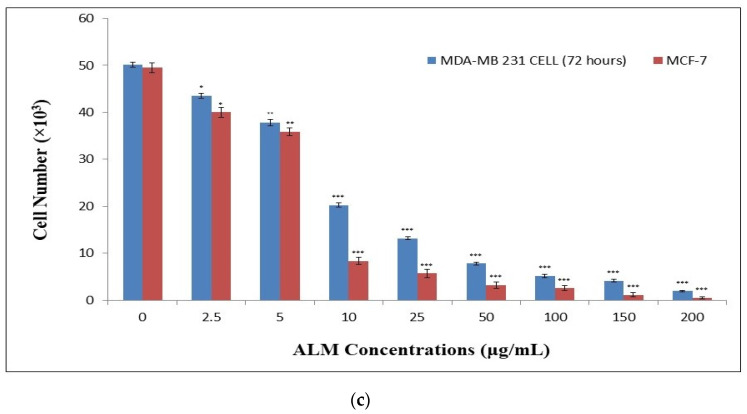
Anti-proliferative effect of *Albizia lebbeck* methanolic (ALM) extract was observed using MTT assay for 24 h (**a**), 48 h (**b**) and 72 h (**c**) incubation period on MDA-MB 231 and MCF-7 cells. Significant differences ((*) *p* < 0.05; (**) *p* < 0.01 and (***) *p* < 0.0001) observed in our graph are applied to all treatments with the same concentration. Data are present as mean ± SEM of at least replicates experiments (*n ≥* 3) and analyzed using one-way ANOVA followed by Tukey’s test. (*) *p* < 0.05; (**) *p* < 0.01 and (***) *p* < 0.0001.

After treatment with ALM extract on both cells, the proliferation assay results revealed anti-proliferative potential in a time- and concentration-dependent manner. Lower concentrations (0, 2.5, 5.0 and 10 μg/mL) did not show any significant difference in the cell proliferation (*p* > 0.05, *n* ≥ 3, Figure 3a–c), but it was observed with concentrations above 10 μg/mL. Anti-proliferative activity of ALM extract increased with increasing concentrations and incubation periods. Significant differences were observed with all concentrations for both MDA-MB 231 and MCF-7 following the 72 h incubation period (*p* < 0.05; *n* ≥ 3, Figure 3c), and that showed that the effect is also time dependent. Similarly, some *Allium* species (*Allium autumnale*) inhibit the proliferation of cells with an increased concentration of the extract and the treatment periods [40]. Furthermore, the study on *Alfaroa mexicana* revealed a high effect on the proliferation of MCF-7 cells and a low on MDA-MB 231 cells [41]. The majority of the concentrations that are toxic to the cells from our studies also revealed an effect on cell proliferation, which could result from cell circle arrest, as pointed out in previous studies [42]. Anti-proliferative potentials of medicinal plants depend on the amount of phytochemical composition of the plants, and the majority of such phytochemicals are flavonoids and phenols [42].

**Figure 4:**
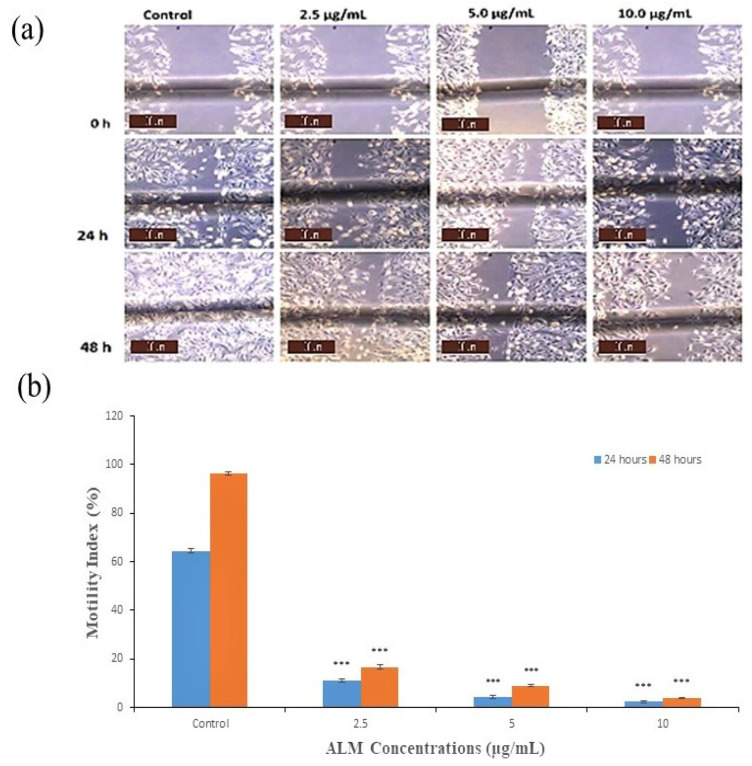
Effect of *A. lebbeck* methanolic (ALM) extract on metastasis of MDA-MB 231 Cell (**a**) Typical phase-contrast light-microscopy (×10) images obtained from wound healing assays of MDA-MB 231 cell following 24 h and 48 h incubation. Scale bar (100 μm) applicable to all panels. (**b**) Bar diagram showing motility index of MDAMB 231 cell following 24 and 48 h incubation ± ALM. Significant differences ((***) *p* < 0.0001) observed in our graph are applied to all treatments with the same concentration. Data are present as mean ± SEM of at least replicates experiments (*n* ≥ 3).

**Figure 5:**
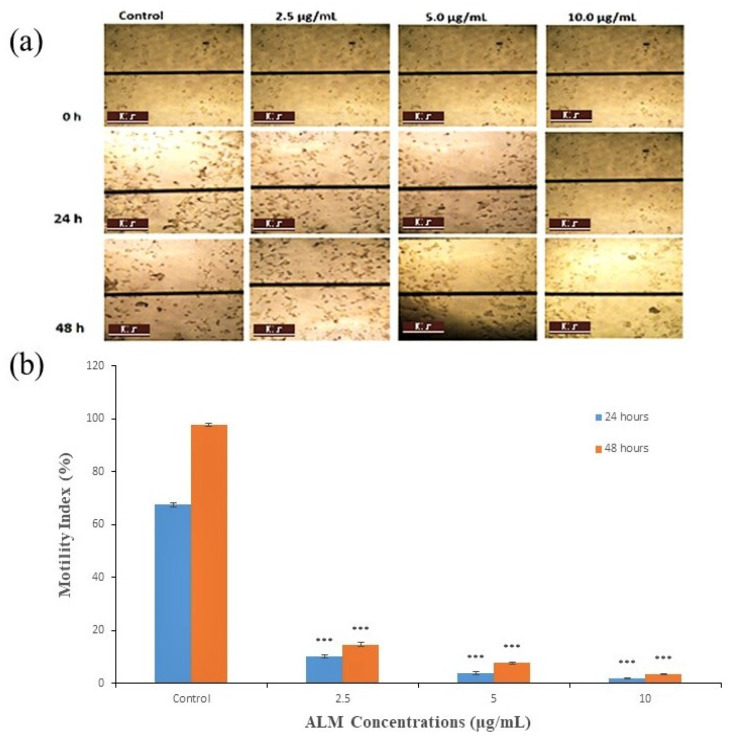
Effect of *A. lebbeck* methanolic (ALM) extract on metastasis of MCF-7 Cell (**a**) Typical phase-contrast light-microscopy (×10) images obtained from wound healing assays of MDA-MB 231 cell following 24 h and 48 h incubation. Scale bar (100 μm) applicable to all panels. (**b**) Bar diagram showing motility index of MDAMB 231 cell following 24 and 48 h incubation ± ALM. Significant differences ((***) *p* < 0.0001) observed in our graph are applied to all treatments with the same concentration. Data are present as mean ± SEM of at least replicates experiments (*n ≥* 3).

Concentrations of 2.5, 5 and 10 μg/mL of ALM extracts that are not toxic and do not affect the proliferation of the cells were used for an anti-metastatic potential study on both cells. In addition, the motility index of MDA-MB 231 cells decreased with increased concentrations of the extract following 24 h incubation period as compared with the treated group (*p* < 0.05; *n* ≥ 3; Figure 4a,b). The best promising motility index was observed with 10.0 μg/mL, followed by 5 μg/mL of the extract. Furthermore, the motility index of MDA-MB 231 increased in all concentrations following 48 h incubation period. The potential of the extract on the lateral motility of MDA-MB 231 depends on the concentration and incubation period, as shown in Figure 4b. Similarly, the motility index of MCF-7 was significantly inhibited with increased concentration of the extract and incubation period when compared with the treated group (*p* < 0.05; *n* ≥ 3; Figure 5a,b), and the highest motility index was observed with 10.0 μg/mL. In addition, the motility index of MCF-7 also increased in all concentrations with an increased incubation period, as in Figure 5b. Metastatic spread is one of the most important steps for the migration of cells [43]. Additionally, quercetin from plant sources interferes with many signal transduction pathways, limits proliferation and metastasis, and brings about apoptosis in breast cancer cells [44]. Inhibition of metastasis using medicinal plants has been reported previously on AT-2 and Mat-LyLu rat prostate cells, and the effect was more on Mat-LyLu, which is a highly metastatic rat prostate cancer cell and is in conformity with the results obtained in our studies [45]. Further silver nanoparticles synthesized using *Ficus ingens* aqueous leaf extract revealed strong anti-metastatic activity against MDA-MB 231 human breast cancer cell line [46].

### 2.2. Artificial Intelligence-Based Models

AI-based models, namely ANFIS and ANN, and a classical MLR were used to predict metastasis in cancer cells treated with extract using our experimental data. Various criteria, such as root mean square error (RMSE), determination coefficient (DC), mean square error (MSE) and correlation coefficient (CC), served as the determinant of the performance indices of the models using MATLAB 9.3 (R2017a). The Levenberg algorithm was applied in ANN modeling using an MSE of 0.0001, 1000 iterations, a momentum coefficient 0.9 and a learning rate of 0.01. The hidden nodes were optimized through the trial-by-error method, whereby the best model was selected. The PE of all the models is shown in Table 2. However, it can be seen that both the classical linear MLR and AI-based models can predict metastasis in MDA-MB 231 and MCF-7 cells upon comparison. Though among the three models, ANFIS shows superior performance compared to ANN and MLR based on the DC, CC, RMSE and MSE. Therefore, the order of the performance of models is ANFIS > MLR > ANN. However, the prediction ability of the DC shows that ANFIS outperformed ANN and MLR and added the PE up to 1% and 0.2%, respectively, in the testing phase for MCF-7, as well as 1% and 0.5% for MDA-MB 231.

**Figure 6:**
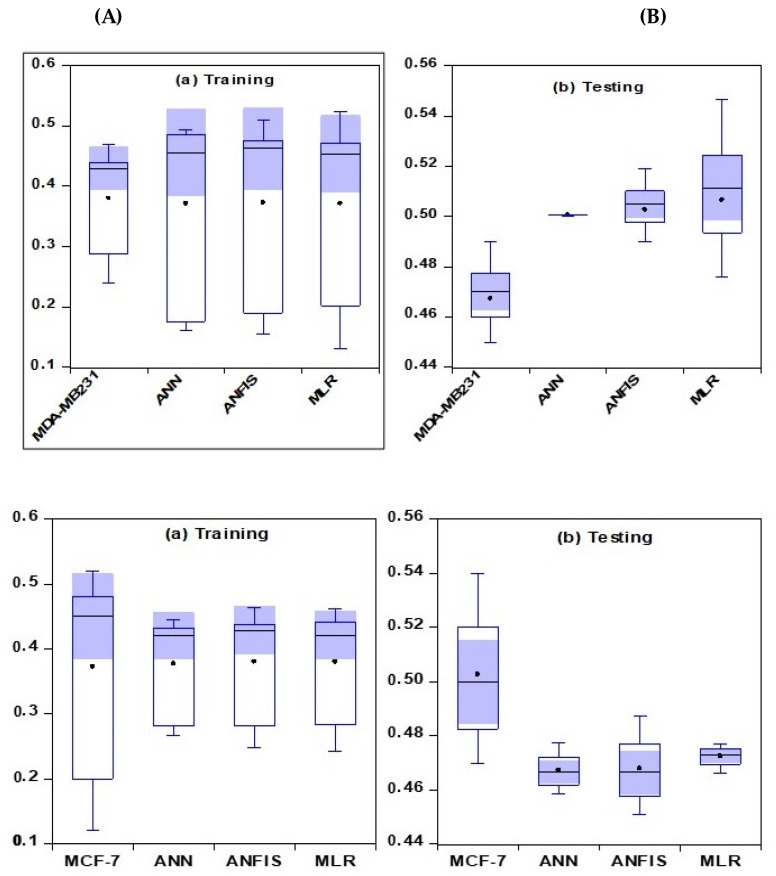
Boxplots showing the extent of the spread between the observed and predicted values (**A**) Training for MDA-MB 231 and MCF-7 cell lines (**B**) Testing for MDA-MB 231 and MCF-7 cell lines.

The comparative analysis of the models represented by boxplots, which give an overview and the statistical summary of the dataset, is illustrated in Figure 6. Based on the representation below, the best model is considered to be the closest one to the measured data using the mean value presented in the plot. The proximity of the measured and the simulated values of the models showed that ANFIS outperformed the two other models and was the best in modeling metastasis in cancer cells for both the testing and training stages.

**Figure 7:**
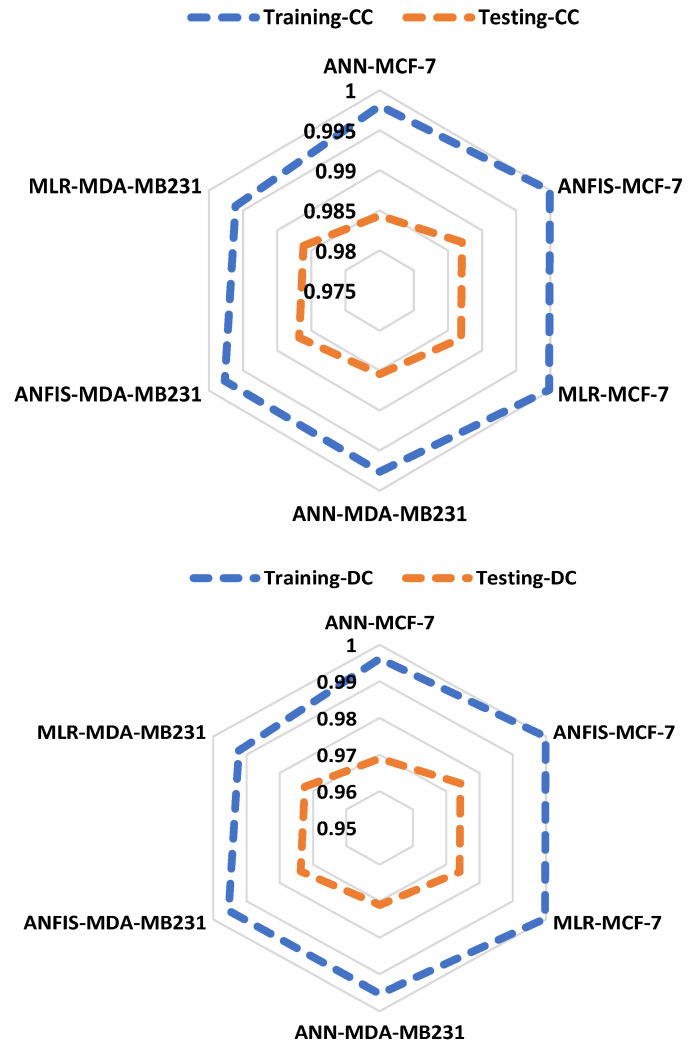
Radar chart for various variation determination coefficients and correlation coefficients.

**Figure 8:**
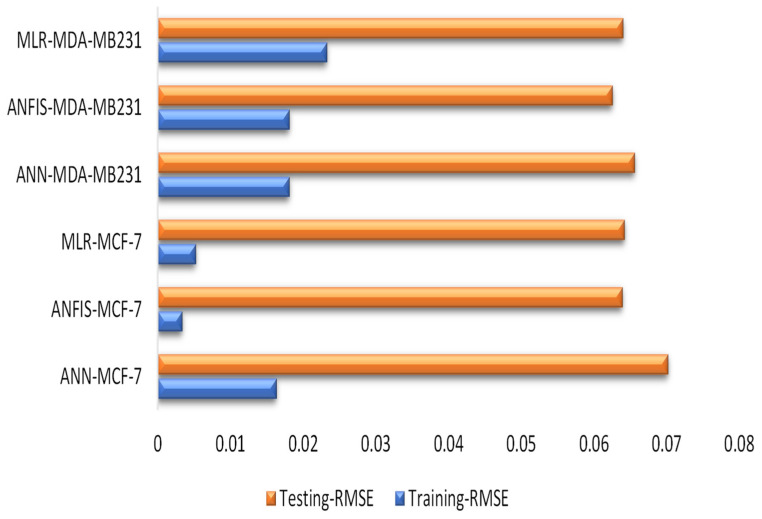
Relative mean square area (RMSA) for predicting MDA-MB 231 and MCF-7 human breast cancer in both the training and testing stages.

The performance accuracy of all the models was shown using a radar chart. The basic application of this chart is to show the minimum and maximum scoring values within a group of datasets, leading to an easy understanding of the performance comparison. Furthermore, the radar chart is used in terms of the determination coefficient and correlation coefficient in both stages (Figure 7). Variations in terms of DC and CC can be observed in Figure 7. In addition, several studies revealed that the performance accuracy of models is attributed to their high DC and CC values [47,48,49,50,51]. Furthermore, Table 2 also reveals the comparative analysis of the models. Our studies have shown that ANFIS outperformed the remaining models by depicting lower values of RMSE in both phases (Figure 8).

**Figure 9:**
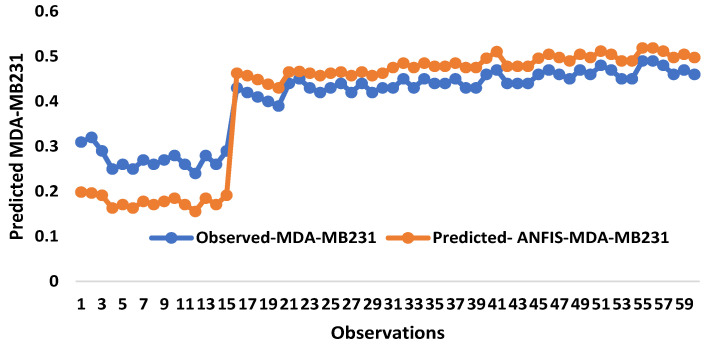

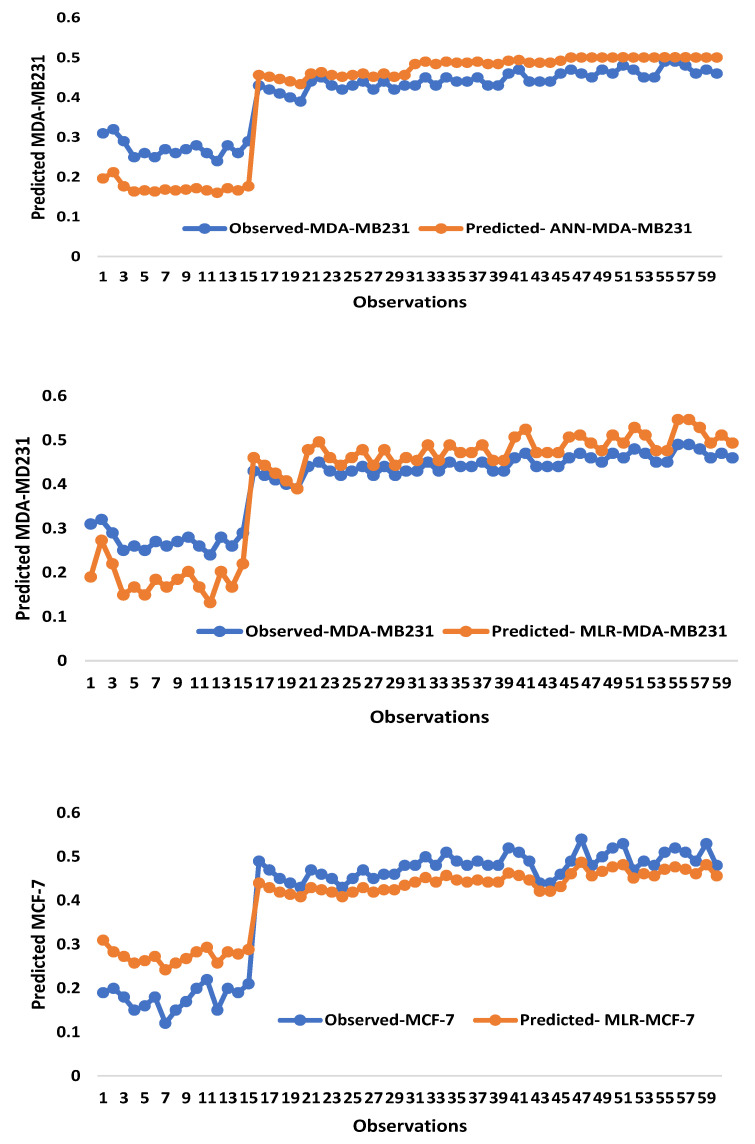

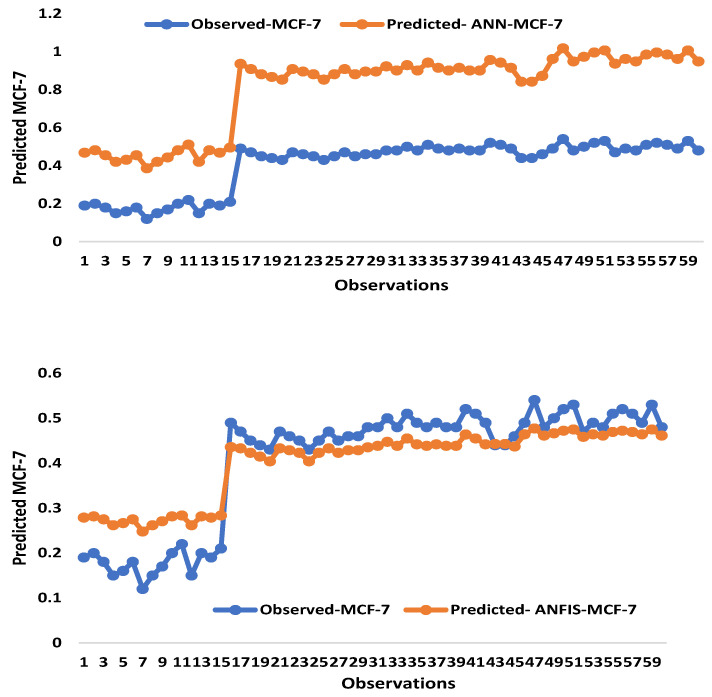
Response surface simulation for treated MDA-MB 231 and MCF-7 human breast cancer in both the training and testing stages.

Simulation response time series graphs were demonstrated for treated cancer cells at the stages (Figure 9). The response proved the measured and the predicted values and the extent of the spread of the values. The response between measured and predicted values are the major determinants of the extent to which the values were distributed, and our data is in conformity with recent findings [52]. Similarly, it can be observed from Figure 8 that traditional and AI-based models captured the oscillating behavior of the measured data, and the plots also revealed that the simulated and measured values are concomitant with each other. In addition, ANFIS (hybrid model) outperformed the two models from the plots, and it captured the majority of the measured data with a high agreement.

## 3. Materials and Methods

### 3.1. Plant Material and Extract Preparation

Stem barks of the plant (*A. lebbeck*) were collected from Gaya, Kano State, Nigeria, during their flowering stage around July 2020 to obtain the *ALM* extract. The plant was authenticated at the Herbarium of the Biology Department, Bayero University Kano, Nigeria. A voucher number BUKHAN187 was assigned to the specimen and deposited at the institute.

Fresh stem barks of *A. lebbeck* were washed carefully with distilled water and air dried in the shade at a specified temperature (25–30 °C). Dried stem barks were then gently pulverized into a coarse powder using a clean, sterile mortar and pestle. For the extraction, 99.9% methanol was used as the solvent for methanolic extract, in which 25 g of the powder was immersed in 250 mL solvents (methanol) in an Erlenmeyer flask for 48 h at 25 °C. The extract was filtered and concentrated through Whatman No. 1 filter paper and a rotary evaporator (Heidolph, Germany), respectively, under reduced pressure. Crude extracts were collected in a sterile vial and labeled, then stored inside a refrigerator prior to the analysis.

### 3.2. Determination of Phytochemical Composition of the Extract

The total phenolic (TPC) and the total flavonoid content (TFC) content of ALM extracts were analyzed spectrophotometrically using standard methods [26,53]. In brief, 0.25 mL of the extract was taken in 15 mL test tubes, followed by the addition of Folin-Ciocalteu reagent (1:10 dilution), incubated for 3 min, 2.0 mL of 7.5% solution of Na_2_CO_3_ was added, incubated and absorbance was recorded at 760 nm using UV visible spectrophotometer (Shimadzu UV-2450) for TPC determination. Similarly, 0.25 mL of 1 mg/mL of ALM extract (1 mg/mL) was taken in a 15 mL test tube, followed by the addition of 0.075 mL of 5% NaNO_2_ solution, 0.15 mL AlCl_3_ (10%) and 0.5 mL of NaOH (1 M), and the absorbance was evaluated at 510 nm with a UV-VIS spectrophotometer for TFC. The results were expressed as gallic acid equivalents (µg GAEs/g dry extract) and mg quercetin equivalent (QE)/g dry extract.

In addition, the organic composition of the extracts was evaluated using GC-MS analysis. The crude extract was prepared in methanol/ethanol (1 mg/mL), filtered using a 0.22 µm syringe filter, and 1 µL injected into Shimadzu, GC-MS-QP2010 plus analyzer. The carrier gas that was used is helium (99.999%) at a constant flow rate of 1 mL/min. Split less injection volume of 0.5ET and injection temperature of 280 °C with a shift line temperature of 300 °C [54]. The initial oven temperature is 50 °C which stands for 2 min, and then gradually rises at the rate of 7 ℃/min. Mass spectra were evaluated at 9.99E2 at a scanning interval of 0.5 s and a full mass scan range from 25 m/z to 1000 m/z using a Quadrupole mass detector. The spectrum obtained was analyzed using the WILLEY7 MS library and revealed the compound.

### 3.3. Cell Lines and Culture Conditions

Human BCa cells, MDA-MB 231 and MCF-7 obtained from Imperial College London were used in the study, and they were approved by the Biotechnology Research Centre Ethical Committee of Cyprus International University (BRCEC2011-01). The cells were cultured in Dulbecco’s Modified Eagle’s Medium (DMEM) (Gibco by Life Technology USA) supplemented with 10% fetal bovine serum (FBS), 2 mM L-glutamine and penicillin/streptomycin. Cell culture standard conditions of 37 °C and 5% CO_2_ were maintained throughout the analysis for all the cells.

### 3.4. Toxicity Assay

Cytotoxicity of ALM extract was ascertained by trypan blue dye exclusion assay on cells [55]. Cells were treated with various concentrations ranging between 0–200 μg/mL and incubated for 24, 48 and 72 h; trypan blue was then added to the 35 mm cell culture dishes. All the experiments were carried out in triplicates.

### 3.5. Proliferation Assay

The proliferation of the treated cells was evaluated using MTT assay as previously described with slight modifications [55]. Culture medium and treatments were replaced every 24 h, and only 1 mL of DMEM medium was used for the treated group. Microplate Reader (ELX 800TM) read the absorbance at 570 nm. All measurements were carried out in triplicates.

### 3.6. Wound-Heal Assay

The effect of various concentrations of ALM extract on lateral motility of the cell was investigated using wound heal assay as described previously by Fraser et al. [55]. Parallel lines were registered on the sterile culture dishes. The MDA-MB 231 cells were seeded at 1 × 10^6^/mL and 5 × 10^5^/mL density per dish for MCF-7 cells; the cells were spread carefully and incubated overnight. Pipette (200 μL) scratches were made using sterile tips along the horizontal lines. After the treatment, wounds were photographed in three stages using a camera mounted on the microscope. Measurement was done using ImageJ software, and the lateral motility was quantified as the motility index as follows:

(1) $$Mo\;I\left(\%\right)=1-\left(\frac{w_{t}}{w_{0}}\right)\times 100$$

Wt represents the wound’s width at a specified period, and W_0_ denotes the initial width at the initial period.

### 3.7. Proposed Methodology

**Figure 10:**
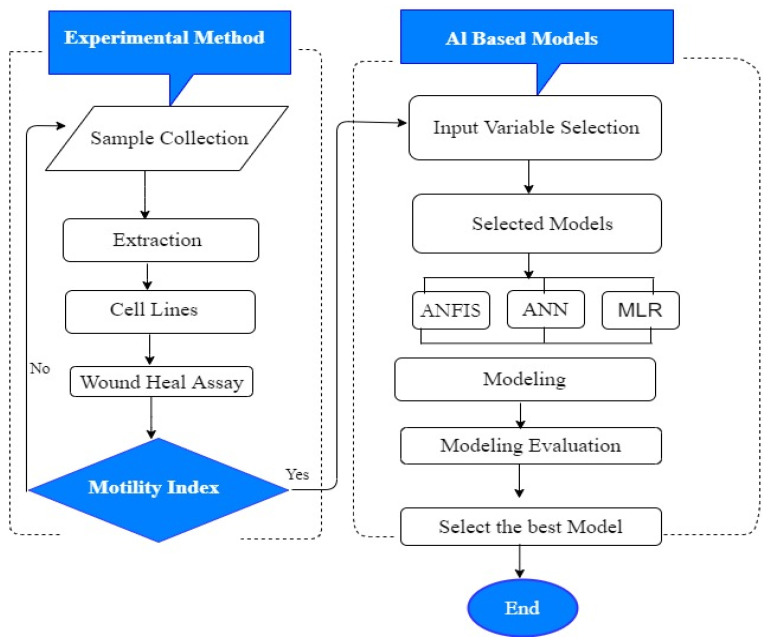
Proposed flowchart of experimental data-driven methods.

Various models were employed as a proposed method to model K’ for in vitro cancer metastasis prediction in highly and weakly metastatic human breast cancer cells. The data used for this data-driven approach were obtained experimentally. The concentration of the extract and motility index are the parameters that we considered in modelling K’, even though other parameters can be considered for the same purpose. Furthermore, three ensemble techniques with single-method output results were used in our study to improve K’s prediction accuracy. A prediction of various parameters needs the use of more than one model, and choosing the desired models is really complex for the predictors and can be resolved through the selection of various assembled models. The significance of our proposed method is to evaluate K’ in metastasis prediction of MCF-7 and MDA-MB 231 cells treated with ALM extract using two various input variables; concentration of the extract and motility index on the cells. The flowchart of our models is shown in Figure 10.

### 3.8. Back Propagation Neural Network (BPNN)

**Figure 11:**
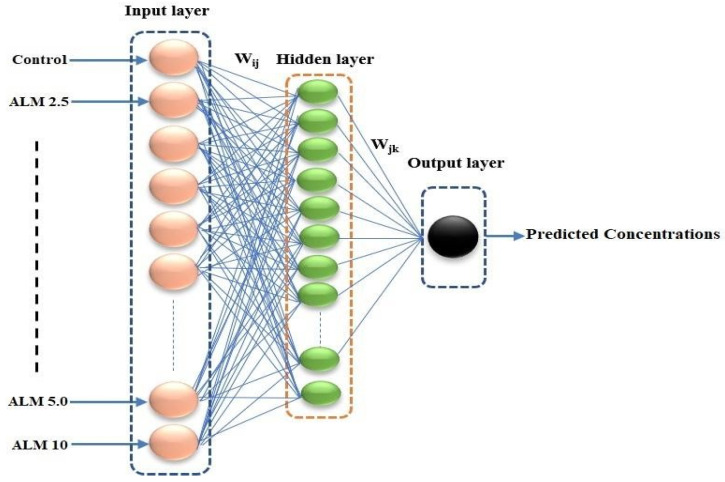
Three layered BPNN algorithms. Peach, green and black colour represent the input, hidden and output layers, respectively.

An ANNs-based model is a commonly used model among all the AI-based models and is designed to function in a similar way to the human brain towards analyzing data and working capacity, and it also discovers relationships in a simple and understandable manner between various sets of data inputs and outputs. Various algorithms in AI but BPNN revealed the most remarkable output because they are instructed with back-propagation algorithm, and they are currently applicable in various fields such as environmental biotechnology, weather and infectious disease forecasting. BPNN depends on hidden input and output layers, as illustrated in Figure 11. Layer nodes (upper and lower) connected with the predicted concentration variables, as shown in Figure 11, lead to the transfer of function and, finally, a transformation of linear space into non-linear space (see Equations (2) and (3)). In addition, ANN modelling directly affects the model efficiency as a result of the functional activities in both layers. Levenberg-Marquardt optimization was used to regulate the network because of its accuracy and efficiency [52].

(2) $$y_{1}=f_{1}\left[\sum_{k=1}^{k}wlkf_{2}\left(\sum_{j=1}^{j}{wlk}_{j}x_{j}+b_{k}\right)+b_{1}\right].$$

(3) $$f_{2}\left(p\right)=\frac{2}{\left(1+e^{-2p}\right)}-1$$

where *x_j_* represents the input variable, *y*_1_ is the output variable, *b*_1_ is the bias, and *f*_1_ and *f*_2_ are the linear and Tran sigmoidal activation functions.

Abbreviations: ALM 2.5, *A. lebbeck* methanolic extract 2.5 μg/mL; ALM 5.0, *A. lebbeck* methanolic extract 5 μg/mL; ALM 10.0, *A. lebbeck* methanolic extract 10 μg/mL.

### 3.9. Adaptive Neuro-Fuzzy Inference System (ANFIS)

ANFIS is a utilized model that enhances the fuzzy Sugeno model, ANN is a single approach, and it has various applications in data analysis, as Jang et al. reported [56]. The application of functions to transform input data into fuzzy values is called fuzzy logic, with values ranging between 0–1. Similarly, ANFIS models also permit modeling considering the following relationship.

Suppose the FIS contains two inputs, ‘x’ and ‘y’, and one output, ‘f,’ a first-order Sugeno fuzzy apply these rules:

(4) $$\mathrm{Rule}\;1:\;\mathrm{if}\;\mu \left(x\right)\;is\;A_{1}\;and\;\mu \left(y\right){\;B}_{1}\;then\;f_{1}=p_{1}x+q_{1}y+r_{1}$$

(5) $$\mathrm{Rule}\;2:\;\mathrm{if}\;\mu \left(x\right)\;is\;A_{2}\;and\;\mu \left(y\right)\;is\;B_{2}\;then\;f_{2}=p_{2}x+q_{2}y+r_{2}$$

$A_{1}$,$B_{1},A_{2},B_{2}$ parameters are membership functions for x and y inputs

$p_{1},q_{1},r_{1,}p_{2},q_{2},r_{2,}$ are the outlet functions.

### 3.10. Multilinear Regression (MLR)

Generally, linear regression can be classified as multiple and simple. The choice of either multiple or linear regression type has to do with the target of the prediction; in simple linear regression (SLR), the comparison is between a single predictor as well as a single variable, but in multiple linear regression (MLR), the correlation is between two or more predictors and with a single variable [57]. In the MLR model, the values from the independent are related to the dependent variables as well, and the data are in two subsets of training and tests [58].

Usually, MLR typically involves estimating the degree of correlation between a single response variable and two or more predictors. The overall expression of MLR is presented in Equation (7).

(6) $$y=b_{0}+b_{1}x_{1}+b_{2}x_{2}+\ldots b_{i}x_{i}$$

where is the predictor, $b_{0}$ is the regression constant and $b_{i}$ is the coefficient of the *i*.

(7) $$R2=1-\frac{\sum_{j=1}^{N}{\left[{\left(Y\right)}_{obs,j}-{\left(Y\right)}_{com,j}\right]}^{2}}{\sum_{j=1}^{N}{\left[{\left(Y\right)}_{obs,j}-{\overset{-}{\left(Y\right)}}_{obs,j}\right]}^{2}}$$

(8) $$RMSE=\sqrt{\frac{1}{N}}\sum_{j=1}^{N}{\left({\left(Y\right)}_{obs,j}-{\left(Y\right)}_{com,j}\right)}^{2}$$

## 4. Conclusions

Overall, thirty-one bioactive compounds were identified from the ALM extract, which might be responsible for the anti-migratory potential of the plant, and it could be a promising cause for the prevention of cancer metastasis. Furthermore, various concentrations of ALM extract revealed the cytotoxic and anti-proliferative potential and a significant decrease in the motility index of both MDA-MB 231 and MCF-7 cells with increased extract concentrations in concentration and incubation period-dependent manner. The cell lines are further predicted using non-linear AI-based models (artificial neuro-fuzzy inference system (ANFIS), artificial neural network (ANN) and classical multilinear regression (MLR) models. The comparative study of the models revealed that MLR and AI-based models are capable of predicting migration potential in MDA-MB 231 and MCF-7 human breast cancer cell lines treated with various concentrations of ALM extract. However, the prediction ability using the determination coefficient showed that ANFIS outperformed ANN and MLR and increased the PE up to 1% and 0.2%, respectively, in the testing phase for MCF-7, as well as 1% and 0.5% for MDA-MB 231. The outcomes of AI-based models suggested that other computational models, such as wavelet and extreme learning machines, need to be introduced for comparisons.

## Figures and Tables

**Table 1:** Bioactive compounds identified from *Albizia lebbeck* methanolic extract by GC-MS analysis and their biological activities.

S/n	RT	Peak Area	Area %	Compound Detected	Biological Activity
1	13.58	96,734	0.61	Cytidine, N-acetyl-(CAS)	Antimicrobial [28]
2	30.91	236,928	1.49	1-Hexadecanol, 2-methyl-(CAS)	Anti-cancer [29,30]
3	31.61	88,990	0.56	1-Hexadecanol, 2-methyl-(CAS)	Anti-cancer, antioxidant and antimicrobial [29,30]
4	32.31	579,671	3.63	2-Propenoic acid, tetradecyl ester	Anti-tumour, anti-inflammatory, anti-mutagenic, [31]
5	33.32	369,272	2.31	Hexadecanoic acid, 2,3 dihydroxypropyl ester	Anti-tumour [32]
6	36.54	445,820	2.79	Hexadecanoic acid, 2,3-dihydroxypropylester	Antioxidant, antimicrobial [33]
7	38.02	189,611	1.19	QUERCETIN 7,3’,4’-TRIMETHOXY	Anti-tumour, [26] anti-hypertensive [34]
8	38.46	802,577	5.03	Dodecanoic acid, 2,3-bis(acetyloxy)propyl ester	Anti-inflammatory, antibacterial [35]
9	39.29	466,439	2.92	Hexadecanoic acid, 2,3-dihydroxypropyl ester	Anti-tumour [32]
10	39.64	315,914	1.98	Hexadecanoic acid, 2,3-dihydroxypropyl ester	Anti-tumour [32], antioxidant, antimicrobial [33]
11	40.40	220,048	1.38	2-Myristynoyl pantetheine	-
12	41.62	321,977	2.02	QUERCETIN 7,3’,4’-TRIMETHOXY	Anti-hypertensive [34]
13	42.49	1,104,712	6.93	Cyclopropanetetradecanoic acid, 2-octyl-, methyl ester	Antibacterial [35]
14	43.05	561,757	3.52	Hexadecanoic acid, 2,3-dihydroxypropyl ester	Anti-tumour [32], antioxidant, antimicrobial [33]
15	43.70	93,671	0.59	QUERCETIN 7,3’,4’-TRIMETHOXY	Antioxidant, anti-tumour [36] anti-hypertensive [37]
16	43.96	500,990	3.14	TRANS-2-PHENYL-1,3-DIOXOLANE-4-METHYL OCTADEC-9,12,15-TRIENOATE	Anti-cancer [38]
17	44.50	160,315	1.01	Hexadecanoic acid, 2,3 dihydroxypropyl ester	Anti-tumour [32], antioxidant, antimicrobial [33]
18	44.82	102,210	0.64	Hexadecanoic acid, 2,3-dihydroxypropyl ester	Anti-tumour [32], antioxidant, antimicrobial [33]
19	45.52	415,295	2.60	TRANS-2-PHENYL-1,3-DIOXOLANE-4-METHYL OCTADEC-9,12,15-TRIENOATE	Anti-cancer [38]
20	45.75	180,204	1.13	Hexadecanoic acid, 2,3-dihydroxypropyl ester	Anti-tumour [32], antioxidant, antimicrobial [33]
21	46.34	1,532,251	9.61	Dotriacontane	Anti-inflammatory, anti-thrombotic, antiviral [33]
22	47.11	242,196	1.52	“Hexadecanoic acid, 2,3-dihydroxypropyl ester”	Anti-tumour [32], antioxidant, antimicrobial [33]
23	47.40	217,328	1.36	Hexadecanoic acid, 2,3-dihydroxypropyl ester (CAS)	Anti-tumour [32], antioxidant, antimicrobial [33]
24	47.99	252,815	1.58	“Hexadecanoic acid, 2,3-dihydroxypropyl ester”	Anti-tumour [32], antioxidant, antimicrobial [33]
25	48.38	307,595	1.93	QUERCETIN 7,3’,4’-TRIMETHOXY	Antioxidant, anti-tumour [36], anti-hypertensive [37]
26	49.33	2,392,813	15.00	Nonacosane (CAS)	Antimicrobial [35]
27	49.93	109,234	0.68	Octadecanoic acid, 2,3-dihydroxypropyl ester	Anti-proliferative, anti-cancer [32,34]
28	50.48	281,862	1.77	“Hexadecanoic acid, 2,3-dihydroxypropyl ester”	Anti-tumour [32], antioxidant, antimicrobial [33]
29	51.39	766,970	4.81	“Hexadecanoic acid, 2,3-dihydroxypropyl ester”	Anti-tumour [32], antioxidant, antimicrobial [33]
30	51.87	92,228	0.58	“9,12,15-Octadecatrienoic acid, 2-[(trimethylsilyl)oxy]-1-[[(trimethylsilyl)oxy]methyl]ethyl ester, (Z,Z,Z)”	Anti-proliferative, anti-cancer [32,34]
31	52.09	2,502,977	15.69	Heptacosane (CAS)	Antimicrobial [35]

**Table 2:** Result of the ANN, ANFIS and MLR models.

	Training					Testing			
	DC		RMSE	MSE	CC	DC	RMSE	MSE	CC
ANN-MCF-7	0.9962		0.0163	0.0003	0.9981	0.9689	0.0702	0.0049	0.9843
ANFIS-MCF-7	0.9998		0.0033	0.0000	0.9999	0.9742	0.0639	0.0041	0.9870
MLR-MCF-7	0.9996		0.0052	0.0000	0.9998	0.9740	0.0642	0.0041	0.9869
ANN-MDA-MB231	0.9953		0.0181	0.0003	0.9976	0.9711	0.0656	0.0043	0.9854
ANFIS-MDA-MB231	0.9953		0.0181	0.0003	0.9976	0.9737	0.0625	0.0039	0.9868
MLR-MDA-MB231	0.9922		0.0232	0.0005	0.9961	0.9725	0.0640	0.0041	0.9861

## Data Availability

Data is contained within the article.

## Abbreviations

AI: artificial intelligence; ALM: *Albizia lebbeck* methanolic extract; ANN: artificial neural network; ANFIS: adaptive neuro-fuzzy inference system; BCa: Breast cancer; BPNN: Back propagation neural network; CC: Correlation Coefficient; DC: Determination Coefficient; DMEM; Dulbecco’s Modified Eagle’s Medium; FBS: Fetal bovine serum; GAE: Gallic Acid Equivalents; PE: Performance Efficiency; MLR: Multilinear regression analysis; MSE: Mean square error; QE: Quercetin Equivalent; RMSE: Relative mean square error; TPC: Total phenolic Content; TFC: Total Flavonoid Content (TFC) content.
